# Oral health status, self-assessment and risk among tribes and narikuravars of Villupuramdistrict, Tamil Nadu – An epidemiological study

**DOI:** 10.1016/j.jobcr.2025.08.018

**Published:** 2025-08-20

**Authors:** V. Kalaivani, Arthi Balasubramaniam, I. Meignana Arumugham

**Affiliations:** Post Graduate, Department of Public Health Dentistry, Saveetha Dental College & Hospital, Saveetha Institute of Medical and Technical Sciences, Saveetha University, Chennai, No.162, Poonamallee High Road, Chennai, 600077, Tamil Nadu, India

**Keywords:** Irular, Narikuravars, Oral health status, Tribal

## Abstract

**Background:**

Irular and Narikuravars, are the oldest Dravidian ethnic group and a semi-nomadic community people located in Tamil Nadu. Both the indigenous groups are relegated to the margins of society and face limited access to oral health care. Thus, we aimed to assess the normative need by assessing their oral health status and risks of Narikuravar and Irular tribes residing in Villupuram District, Tamil Nadu.

**Method:**

ology: A cross-sectional epidemiological study with convenience sampling technique was conducted for 936 Irular and Narikuravar community people in Marakanam and Valavanur town panchayat of Dindivanam taluk of Villupuram district. Oral health status and self-assessment of oral health and risks was assessed using World Health Organization (WHO) oral health assessment form, self-oral health assessment form (2013).

**Result:**

Narikuravar constitutes 32.8 % whereas Irula's constitute 67.2 % of the study population. Nearly 39.9 % of the study population had no formal schooling. Irular females with no formal schooling had high DMFT scores (3.27 ± 1.60). Also, the prevalence of gingivitis and periodontitis are high in Irular females with no formal schooling (32.3 % and 26 %) with no significant difference from Narikuravars. About 5.2 % of Narikuravar communities had high use of smoking and smokeless tobacco. Irulars consumed high cariogenic diet compared Narikuravars (p = 0.000).

**Conclusion:**

Narikuvar community had more oral lesion compared to Irula's community people. Both communities had high prevalence of dental caries and periodontal disease. Females with no formal schooling of both communities had high caries experience, periodontitis.

## Graphical abstract

**Figure:**
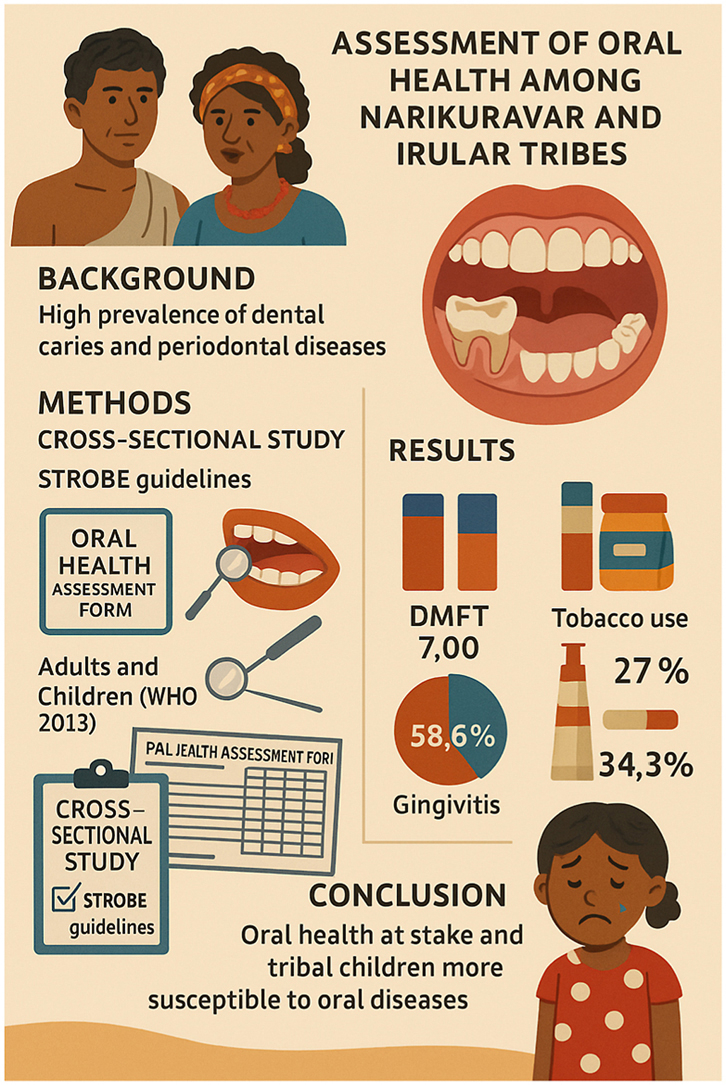

## 1 Introduction

Tribal population in India constitutes about 8.6 % of the total population as per 2011 census.^1^ The scheduled tribes comprise 2.8 % of the urban population and 11.3 % of the rural national population.^2^ Primitive tribal groups are more susceptible to disease, and their misery is made worse by factors such as poverty, illiteracy, ignorance about disease leading to unpleasant environments, inadequate sanitation, lack of access to good drinking water.^3^ Accessibility to health care becomes impossible due to lack of awareness, lack of transport as they live in remote areas, poverty, and non-availability of essential drugs. Although global oral health has seen significant progress, many challenges remain, especially within underprivileged and underserved communities. In Tamil Nadu, there are 36 Tribal communities contributing to 1.10 % of the total population as per the 2011 census.^4^ The Irular and Narikuravar are one among the few tribes who are still backward in terms of social, economic, political, and educational considerations.

Studies have been done to assess the oral health status of different tribes in India. The Periodontal status of Koya and Lambda in Telangana has been studied, both the groups have compromised periodontal status.^5^ The oral health status of tribes residing in Chamarajanagar district, Karnataka has a high prevalence of dental caries and periodontal diseases.^6^ A study done on Tribals in Northern Bhubaneswar, Odisha showed a high prevalence of dental caries and periodontal disease.^7^ Iruliga tribals in Karnataka were using chew sticks instead of toothbrushes and there is a low prevalence of periodontal disease.^8^ A study on Paniya tribes in Kerala showed nearly 90 % of the population has a habit of pan chewing with/without Tobacco.^9^ A study on Gypsy people reveals that 60 % of the study population was using tobacco.^10^

As only a few studies have been done in the Narikuravar and Irular communities, it is important to know about their oral health status and health beliefs to plan a program which can improve their oral health. Assessing oral health status, oral hygiene practices and treatment needs of Indigenous people helps to know their attitude and awareness towards dental problems. This epidemiological study may help in understanding of political, social, biological factors exacerbating the oral diseases among tribes and narikuravars. Also, they help in planning, stating oral health policies and implement adequate accessible health services to improve the oral health state of tribal population. Thus, this study aims to assess the oral health status and investigate self-assessment of oral health and risk of Narikuravar and Irular Tribes residing in Villupuram District, Tamil Nadu.

## 2 Materials and METHODS

### 2.1 Study design, participants and setting

This study was a cross-sectional epidemiological study which followed Strengthening the Reporting of Observational Studies in Epidemiology (STROBE) guidelines. A convenience sampling technique was adopted which selected all the people of tribal villages of Villupuram district present on the days of examination. However, by making frequent visits to the villages, total population of the village panchayat was examined. The study was conducted in Marakanam and Valavanur town panchayat of Tindivanam Taluk. This study was carried out over a six-month period, from December 2022 to July 2023.

By lottery method, Tindivanam taluk was selected from eight taluks of Villupuram district. Of 68 villages in the taluk, Marakanam and Valavanur panchayat was picked using lottery method. From the selected town panchayats, people belonging to the Narikuravar and Irular community who were willing to participate were included in the study.

### 2.2 Sample size

No sample size was calculated for this epidemiological study, since all tribal population residing in the Markanam and Valvanur panchayat, willing to participate were included.

### 2.3 Ethical clearance

The study received approval from the institutional review board of the author's institution (Scientific Review Board Number: SRB/SDC/PHD-2101/22/031). The goal of the study has been explained to the participants before obtaining the written consent form. Consent forms were read aloud to those who had trouble reading them. The Declaration of Helsinki was followed when conducting the study. Those who were seriously ill at the time of the study and did not provide informed consent were excluded. In each taluk, permission to conduct the study in the villages had been sought from the relevant authorities.

### 2.4 Data collection and survey instruments

Demographic details of the participants were obtained followed by the Oral Health Assessment Form for Adults and Children, WHO 2013, which was used to record the self-oral health assessment and risk. The Questionnaire was provided both in English and vernacular language. The investigator recorded the self-assessment form after asking the questions for those who were not able to read. Dental health assessments of participants were conducted using a plain mouth mirror and a Community Periodontal Index (CPI) probe in accordance with WHO guidelines. The right amount of natural light was used to conduct a Type III clinical examination. The instruments were sterilised following standard protocol at the study settings. A door-to-door survey with oral examination was carried out. To ensure consistency in diagnosis among the study participants, a single examiner was trained and calibrated in the Department of Public Health Dentistry of authors University. The investigator herself with the assistance of recorders carried all the clinical examinations. The Kappa value for intra-examiner reliability was 0.8. Age of participants were group as 2–5 years, 6–12 years, 13–15 years, 16–34 years, 35–44 years, 45–64 years, 65–74 years, and 75–85 years for statistical analysis.

### 2.5 Statistical analysis

The data was analyzed using the SPSS software package, version 23, developed by IBM Corporation in Chicago, IL, USA. Descriptive statistics, including percentages, means, and standard deviations, were utilized for demographic and clinical details. A chi-square test to compare the categorical data and One-Way ANOVA with post-hoc test, independent *t*-test to compare the continuous data was employed. The standard for significance was fixed at p-value ≤0.05.

## 3 Results

In our study, among the study population, 40.8 % were males and 59.2 % were females. About 32.8 % of the study population were Narikuravars and 67.2 % were Irulars. Nearly 39.9 % of the study population had no formal schooling, 40.4 % of the population had completed primary schooling, and 7.7 % and 6.8 % had finished secondary and higher secondary education. Most of the study participants (89.1 %) used tooth brush and tooth paste to maintain their oral hygiene. There was significant difference in the distribution of age group, gender, education, and oral hygiene practices among the study participants (Table 1).

**Table 1:** Demographic details of the study participants.

Variable	Sub-variable	N (%)	p-value
**Age**	2–5 years	21 (2.2 %)	0.000
	6–12 years	78 (8.3 %)	
	13–15 years	26 (2.8 %)	
	16–34 years	367 (39.2 %)	
	35–44 years	154 (16.5 %)	
	45–64 years	214 (22.9 %)	
	65–74 years	58 (6.2 %)	
	75–85 years	18 (1.9 %)	
**Sex**	Male	382 (40.8 %)	0.000
	Female	554 (59.2 %)	
**Ethnicity**	Irular	629 (67.2 %)	0.000
	Narikuravar	307 (32.8 %)	
**Education**	No formal schooling	374 (39.9 %)	0.000
	Less than primary school	362 (38.7 %)	
	Primary school	16 (1.7 %)	
	Secondary	72 (7.7 %)	
	Higher secondary	64 (6.8 %)	
	Degree	40 (4.3 %)	
	Postgraduate	8 (0.9 %)	
**Oral hygiene aids**	Tooth brush and tooth paste	834 (89.1 %)	0.000
	Finger and brick powder/charcoal/ash	102 (10.9 %)	

A significant high mean DMFT was found among the females of 65–74 years and 75–85 years of age group (7.00 ± 4.747; 9.33 ± 5.096) (Table 2) Based on ethnicity, Irulars had 1.88 ± 1.2 and Narikurava's had 1.75 ± 1.18 with no significant difference among them (p > 0.05). Statistically significant high mean DMFT was evident among the study participants with no formal schooling (3.27 ± 1.60) (Table 3).

**Table 2:** Sub group analysis of Decay, Missing and Filled Teeth (DMFT) scores among the study participants.

Age	Gender	N	Mean ± SD	p-value
2–5 years	Male	8	2.00 ± 0.000	0.000
	Female	13	6.00 ± 0.834	
6–12 years	Male	32	4.67 ± 2.929 3.17 ± 1.078	
	Female	46		
13–15 years	Male	9	1.50 ± 0.516 3.00 ± 3.592	
	Female	17		
16–34 years	Male	129	5.10 ± 2.483 6.80 ± 2.241	
	Female	238		
35–44 years	Male	68	6.00 ± 2.691 5.27 ± 3.164	
	Female	86		
45–64 years	Male	104	5.50 ± 3.805 6.54 ± 2.352	
	Female	110		
65–74 years	Male	25	6.00 ± 4.126 7.00 ± 4.747	
	Female	33		
75–85 years	Male	7	5.00 ± 4.370 9.33 ± 5.096	
	Female	11

**Table 3:** Comparison of mean DMFT values among the study participants based on demographic details.

Variable	Sub-variable	N	Mean ± S.D	P Value
**Ethnicity**	Irular	629	1.88 ± 1.191	0.514
	Narikuravar	307	1.75 ± 1.180	
**Education**	No formal schooling	374	3.27 ± 1.60	0.000
	Less than primary school	362	1.94 ± 1.34	
	Primary school completed	16	2.00 ± 1.03	
	Secondary school completed	72	2.22 ± 1.32	
	High school completed	64	1.50 ± 0.71	
	College/university completed	40	1.20 ± 0.40	

High prevalence of gingivitis (>55 %) and periodontitis (>70 %) was seen among 65–85 years of age group with a significant difference. Prevalence of gingivitis and periodontitis gradually increased from 35 to 44 years of age group. Males had a higher prevalence of gingivitis (27.7 %) and periodontitis (20.9 %) than females with no significance. Prevalence of gingivitis was high among Irulars and prevalence of periodontitis was high among Narikuravars with a significant difference (Table 4).

**Table 4:** Distribution of periodontitis among the study population.

Variable	Sub-variable	Gingivitis N (%)		P value	Periodontitis N (%)		P value
		Present	Absent		Present	Absent	
**Age**	2–5 years	3 (14.3 %)	18 (85.7 %)	0.000	0	21 (100 %)	0.000
	6–12 years	27 (34.6 %)	51 (65.4 %)		0	78 (100 %)	
	13–15 years	6 (23.1 %)	20 (76.9 %)		0	26 (100 %)	
	16–34 years	40 (10.9 %)	327 (9.1 %)		48 (13.1 %)	319 (86.9 %)	
	35–44 years	32 (20.8 %)	122 (79.2 %)		24 (15.6 %)	130 (84.4 %)	
	45–64 years	80 (37.4 %)	134 (62.6 %)		64 (29.9 %)	150 (70.1 %)	
	65–74 years	34 (58.6 %)	24 (41.4 %)		42 (72.4 %)	16 (27.6 %)	
	75–85 years	10 (55.6 %)	8 (44.4 %)		0 (0 %)	18 (100 %)	
**Sex**	Male	106 (27.7 %)	276 (72.3 %)	0.090	80 (20.9 %)	302 (79.1 %)	0.220
	Female	126 (22.7 %)	428 (77.3 %)		98 (17.7 %)	456 (82.3 %)	
**Ethnicity**	Irular	169 (26.9 %)	460 (73.1 %)	0.021	106 (16.9 %)	523 (83.2 %)	0.001
	Narikuravar	63 (20.5 %)	244 (79.5 %)		72 (23.5 %)	235 (76.5 %)	
**Education**	No formal schooling	82 (32.3 %)	172 (67.7 %)	0.000	66 (26 %)	188 (74 %)	0.000
	Less than primary school	74 (20.4 %)	288 (79.6 %)		96 (26.5 %)	266 (73.5 %)	
	Primary school	7 (43.8 %)	9 (56.2 %)		8 (50 %)	8 (50 %)	
	Secondary	24 (33.3 %)	48 (66.7 %)		8 (11.1 %)	64 (88.9 %)	
	Higher secondary	26 (40.6 %)	38 (59.4 %)		0	64 (100 %)	
	Degree	16 (40 %)	24 (60 %)		0	40 (100 %)	
	Postgraduate	2 (30 %%)	6 (70 %)		0	8 (100 %)	

About 4.4 % of the study population of 16–34 years of age group had leukoplakia and 5.2 % of 35–44 years had tobacco pouch keratosis. Males (4.2 %) are affected more compared to females (2.9 %) with no significance. Leukoplakia was more prevalent among Narikuravar community with asignificant difference from Irular community. Also, people who had less than primary schooling (6.6 %) had high prevalence of Leukoplakia with a significant difference (p < 0.05) (Table 5).

**Table 5:** Distribution of oral mucosal lesions among the study population.

Variable	Sub-variable	N (%)			p- value
		No lesion	Leukoplakia	Tobacco pouch keratosis	
**Age**	2–5 years	21 (100 %)	0	0	0.000
	6–12 years	78 (100 %)	0	0	
	13–15 years	26 (100 %)	0	0	
	16–34 years	351 (95.6 %)	16 (4.4 %)	0	
	35–44 years	138 (89.6 %)	8 (5.2 %)	8 (5.2 %)	
	45–64 years	206 (96.3 %)	8 (3.7 %)	0	
	65–74 years	58 (100 %)	0	0	
	75–85 years	18 (100 %)	0	0	
**Sex**	Male	366 (95.8 %)	16 (4.2 %)	0	0.036
	Female	530 (95.7 %)	16 (2.9 %)	8 (1.4 %)	
**Ethnicity**	Irular	605 (96.2 %)	16 (2.5 %)	8 (1.3 %)	0.016
	Narikuravar	291 (94.8 %)	16 (5.2 %)	0	
**Education**	No formal schooling	358 (95.8 %)	8 (2.1 %)	8 (2.1 %)	0.000
	< primary school	338 (93.4 %)	24 (6.6 %)	0	
	Primary school	16 (100 %)	0	0	
	Secondary	72 (100 %)	0	0	
	Higher secondary	64 (100 %)	0	0	
	Degree	40 (100 %)	0	0	
	Postgraduate	8 (100 %)	0	0	

Solid sugars are taken more every day by people of the age group 13–15 years (100 %), and liquid sugars are taken more by people between 6 and 44 years of age every day with a significant difference. Males and females were taking solid and liquid sugars approximately equally. (Table 6). Smoking was more prevalent in the age group of 15–44 years and all of them were males. Chewable tobacco was predominantly used by people of the age group 16–64 years with a significant difference. (Table 7).

**Table 6:** Distribution of dietary habit practices among the study population.

Variable	Sub-variable	Sugar							
		Solid				Liquid			
		Everyday	Several times a week	Once a week	P value	Everyday	Several times a week	Once a week	p- value
**Age**	2–5 years	5 (23.8 %)	0	16 (76.2 %)	0.000	2 (9.5 %)	5 (23.8 %)	14 (66.7 %)	0.000
	6–12 years	65 (83.3 %)	11 (14.1 %)	2 (2.6 %)		27 (34.6 %)	34 (43.6 %)	17 (21.8 %)	
	13–15 years	26 (100 %)	0 (0 %)	0 (0 %)		5 (19.2 %)	7 (26.9 %)	14 (53.8 %)	
	16–34 years	313 (85.3 %)	36 (9.8 %)	18 (4.0 %)		115 (31.3 %)	139 (37.9 %)	113 (30.8 %)	
	35–44 years	121 (78.6 %)	15 (9.7 %)	18 (11.7 %)		53 (34.4 %)	60 (39 %)	41 (26.6 %)	
	45–64 years	126 (58.9 %)	16 (7.5 %)	72 (33.6 %)		65 (30.4 %)	55 (25.7 %)	94 (43.9 %)	
	65–74 years	41 (70.7 %)	12 (20.7 %)	5 (8.6 %)		0	0	58 (100 %)	
	75–85 years	4 (22.2 %)	1 (5.6 %)	13 (72.2 %)		0	0	18 (100 %)	
**Sex**	Male	288 (75.4 %)	38 (9.9 %)	56 (14.7 %)	0.000	108 (28.3 %)	125 (32.7 %)	149 (39 %)	0.002
	Female	413 (74.5 %)	53 (9.6 %)	88 (15.9 %)		159 (28.7 %)	175 (31.6 %)	220 (39.7 %)	
**Ethnicity**	Irular	478 (76 %)	57 (9.1 %)	94 (14.9 %)	0.000	182 (28.9 %)	198 (31.5 %)	249 (39.6 %)	0.061
	Narikuravar	223 (72.6 %)	34 (11.1 %)	50 (16.3 %)		85 (27.7 %)	102 (33.2 %)	120 (39.1 %)	
**Education**	No formal schooling	271 (72 %)	43 (11 %)	60 (17 %)	0.000	105 (27.5 %)	110 (31.5 %)	159 (41 %)	0.000
	Less than primary school	279 (77.1 %)	31 (8.6 %)	52 (14.4 %)		103 (28.5 %)	113 (31.2 %)	146 (40.3 %)	
	Primary school	11 (68.8 %)	1 (6.3 %)	4 (25 %)		6 (37.4 %)	5 (31.3 %)	5 (31.3 %)	
	Secondary	52 (72.2 %)	9 (12.5 %)	11 (15.3 %)		23 (31.9 %)	29 (40.3 %)	20 (27.8 %)	
	Higher Secondary	50 (78.1 %)	4 (6.3 %)	10 (15.6 %)		14 (21.9 %)	28 (43.8 %)	22 (34.3 %)	
	Degree	31 (77.5 %)	3 (7.5 %)	6 (15 %)		12 (30 %)	13 (32.5 %)	15 (37.5 %)	
	Postgraduate	7 (87.5 %)	0	1 (12.5 %)		4 (50 %)	2 (25 %)	2 (25 %)

**Table 7:** Distribution of adverse habit practices among the study population.

Variable	Sub-variable	Adverse habits									
		Smoking					Chewing				
		No habit	Everyday	Several times a week	Once a week	p- value	No habit	Everyday	Several times a week	Once a week	p-value
**Age**	2–5 years	21 (100 %)	0	0	0	0.000	21 (100 %)	0	0	0	0.000
	6–12 years	78 (100 %)	0	0	0		78 (100 %)	0	0	0	
	13–15 years	23 (88 %)	0 (0 %)	1 (4 %)	2 (8 %)		24 (92 %)	0	1 (4 %)	1 (4 %)	
	16–34 years	278 (76 %)	41 (11 %)	29 (8 %)	19 (5 %)		251 (68 %)	36 (10 %)	52 (14 %)	28 (8 %)	
	35–44 years	97 (63 %)	21 (13 %)	12 (8 %)	24 (16 %)		89 (58 %)	34 (22 %)	13 (8 %)	18 (12 %)	
	45–64 years	144 (67 %)	37 (17 %)	19 (9 %)	14 (7 %)		132 (62 %)	45 (21 %)	14 (7 %)	23 (10 %)	
	65–74 years	43 (74 %)	7 (12 %)	3 (5 %)	5 (9 %)		28 (48.3 %)	18 (31 %)	8 (13.7 %)	4 (7 %)	
	75–85 years	13 (72 %)	3 (16 %)	1 (6 %)	1 (6 %)		6 (33.3 %)	9 (50 %)	2 (11.1 %)	1 (5.6 %)	
**Sex**	Male	143 (37 %)	109 (29 %)	65 (17 %)	65 (17 %)	0.305	158 (41.3 %)	101 (27.4 %)	66 (17.3 %)	57 (14 %)	0.286
	Female	554 (100 %)	0	0	0		471 (85 %)	41 (7.5 %)	24 (4.3 %)	18 (3.2 %)	
**Ethnicity**	Irular	463 (73 %)	73 (12 %)	51 (8 %)	42 (7 %)	0.177	413 (65.7 %)	80 (12.7 %)	76 (12 %)	60 (9.6 %)	0.850
	Narikuravar	234 (76 %)	36 (12 %)	14 (5 %)	23 (7 %)		216 (70.4 %)	62 (20.2 %)	14 (4.6 %)	15 (4.8 %)	
**Education**	No formal schooling	292 (77 %)	43 (12 %)	21 (6 %)	18 (5 %)	0.000	281 (77 %)	61 (16 %)	17 (4 %)	15 (3 %)	0.000
	Less than primary school	293 (82 %)	28 (7 %)	15 (4 %)	26 (7 %)		269 (74.3 %)	34 (9.3 %)	33 (9.1 %)	26 (7.3 %)	
	Primary school	7 (44 %)	4 (25 %)	3 (19 %)	2 (12 %)		6 (37.5 %)	6 (37.5 %)	3 (19 %)	1 (6 %)	
	Secondary	37 (51 %)	13 (18 %)	15 (21 %)	7 (10 %)		21 (29.1 %)	16 (22.2 %)	21(29.2 %)	14 (19.5 %)	
	Higher Secondary	41 (64 %)	8 (12.5 %)	7 (11 %)	8(12.5 %)		28 (43.8 %)	15 (23.4 %)	9 (14 %)	12 (18.8 %)	
	Degree	22 (55 %)	12 (30 %)	4 (10 %)	2 (5 %)		19 (47.5 %)	8 (20 %)	7 (17.5 %)	6 (15 %)	
	Postgraduate	5 (62.5 %)	1 (12.5 %)	0	2 (25 %)		5 (62.5 %)	2 (25 %)	0	1 (12.5 %)	

## 4 Discussion

The Irulas and Narikuravas are typical of tribal people in that they possess their unique eating patterns, which are influenced by their cultural and social beliefs. Their oral hygiene practices, eating pattern along with educational and economic status influences their oral and general health. Lack of awareness about available treatment options and their residency in the remote making them difficult to access and utilize health care. This worsens the condition and increases the prevalence of oral disease in tribal communities. Individual thinking and behaviour are linked to their literacy rate which also contributes to their oral health maintenance and utilization of health care. This study would advance our understanding and comparison of oral status of these indigenous people by providing a thorough overview of the studies that have been done on the tribal community.

The present study was carried out on Irulas and Narikuravas residing in the Villupuram District, Tamil Nadu. The study participants were divided into eight age groups, that is, 2–5 years, 6–12 years, 13–15 years, 16–34 years, 35–44 years, 45–64 years, 65–74 years and 75–85 years. The present study evidently showed that a majority (89.1 %) of Irulas and Narikuravas tribals routinely used tooth brush and toothpaste to clean their teeth, whereas study done on Santhal tribes in Dhanbad, Jharkhand used twigs to clean their teeth (74.3 %) and finger (7.9 %), only 17.8 % were using a toothbrush.^11^ In Konda Reddy tribals nearly 93.6 % population used twigs to clean their teeth, remaining 6.3 % used combination of, finger, twig and toothbrush with toothpaste and charcoal.^12^ The present study reported that 89.1 % of the tribes and narikuravars use tooth brush and tooth paste as their oral hygiene aids. In consistent with this study, a study reported 85.7 % of Irular children in Nilgiri hills of Tamil Nadu brushed their teeth using tooth brush and tooth paste.^13^

A study by Mandel et al.^14^ showed lesser DMFT/deft scores in santal tribes compared to our study findings, However, the DMFT/deft scores in santal tribes by Kumar et al.^7^ was found to be consistent with our findings. The mean DMFT/deft score for 13–15-year-old males and females were 1.50 ± 0.516 and 3.00 ± 3.592 respectively which is higher than school-going children aged 12 and 15 years in the Bhopal district (Male & Female, 0.54 ± 1.06 and 0.63 ± 1.05, 0.52 ± 1.24 and 0.67 ± 1.33 respectively).^15^ These findings indicate that the oral disease burden is higher in tribal children compared to non-tribal children, highlighting the urgent need for policies to reduce the prevalence of dental caries. A similar study among the Irulars and Narikurvars of Vellore district showed a high DMFT score in Irular communities (6.58) than Narikuravars (5.30).^16^ Another study among Kanikaran tribes in Tamil Nadu showed a high mean DMFT among 19–30 years females (4.80 ± 2.70), like the present study (6.80 ± 2.241).^17^ The prevalence of dental caries varies among the different tribes, emphasizing the need for thorough oral health assessments in tribal areas. Early treatment can significantly reduce the disease burden in children. The mean DMFT score was found to be high among the tribal female geriatrics (7.00 ± 4.747). Similar, to this finding, a study reported mean DMFT score of 18.3 ± 8.12 among the tribal geriatrics of Nilgiri hills of Tamil Nadu, impacting their psychosocial and functional domains of oral health related quality of life.^18^

The prevalence of gingivitis was higher among people aged 65–74 years (58.6 %, n = 34) followed by 75–85 years (55.6 %, n = 10), whereas tribes from Bhubaneswar, Odisha had a higher prevalence of gingivitis in the age group of 35–44 years.^7^ Gender plays no role in the prevalence of gingivitis among the study population. Based on education, those who have no primary schooling had a higher prevalence of gingivitis (43.8 %). This high prevalence of gingivitis among people with no primary schooling may be due the lack of awareness.

Periodontitis increased with age in our study population, people of age 75–85 years had the highest prevalence (88.9 %) which was high compared to a study done in Kerala. Only 23.2 % of tribals above the age of 60 years in Wayanad, Kerala had periodontitis.^19^ In tribes of Odisha, deep pockets were common in age group of 65–74 years shallow pockets were common among the age of 35–44 years.^7^ Similarly, among Paniya tribes, 14 % had shallow pockets among aged 35–44 years, 4.2 % in the age group of 65–74 years and deep pockets were common in 20 % of 65–74 years of age and only 3.9 % affected in 35–44 years of age.^9^ In the Koya and Lambada tribes, shallow pockets were high, no notable differences were observed between these groups.^5^

Most of the population was free of oral mucosal lesions, and nearly 32 % had Leukoplakia, of which 4.2 % were males and 2.9 % were females. Oral lesions were prevalent between the ages of 16 and 64 years. In Santal tribes, only 2.7 % had Leukoplakia^14^ which was lesser compared to that of tribes living in Northern Bhubaneswar, Odisha (6.73 %).^7^ Tribal patients above 60 years in Wayanad, Tamil Nadu had a higher prevalence of Oral lesions than other tribes.^19^, ^20^, ^21^ A cross-sectional study conducted among nomadic population of Jawadhu hills of Tamil Nadu found a high prevalence of pouch keratosis (16.9 %).^22^ However, the present study reported a 5.2 % prevalence of tobacco pouch keratosis among the Irulars.

Tobacco usage was high among Irulas compared to Narikuravas, smoking (Irulas 27 %, Narikuravas 23 %) and smokeless tobacco (Irulas 34.3 %, Narikuravas 29.6 %). Prevalence of Tobacco usage (smokeless and smoking) was high among Males (smoking 63 %, smokeless Tobacco 58.7 %). People aged 35–64 years and who had done primary and secondary school have a higher prevalence of smoking habit, whereas smokeless tobacco usage was more prevalent among 65–85 years. Tribals in Odisha had a higher prevalence of tobacco usage than other tribals, nearly 77.31 % of the population used Paan, and 39.57 % of the population used Khaini followed by Gutkha and Cigarette/Bidi (26.80 % and 39.57 %).^7^ This study showed a higher sugar consumption in the form of solids among the 13–15 years of age group, in consistency with the results of the previous study among South Indian and Malaysian children.^23^

A study which evaluated the knowledge, attitude, and utility of government health schemes among the tribal gypsies in Tamil Nadu, found that 14.4 % of them were not aware about the schemes and 68.2 % felt there was no one to guide them in health schemes.^24^ This might be the reason for the high prevalence of oral diseases among the tribes and narikuravars of the state in the present study, which needs to addressed.

A limitation of this study is its cross-sectional design, which captures only a single moment in time, reflecting the oral health status and behaviors of the Narikuravar and Irular communities. This design limits the ability to establish causality between observed oral health outcomes and the various demographic or behavioral factors. Additionally, the study is geographically confined to the Villupuram District in Tamil Nadu, which may limit the generalizability of the findings to other tribal populations in India with different environmental, social, or cultural conditions. Furthermore, the reliance on self-reported data, particularly in a population with low literacy levels, may introduce information and interviewer biases and inaccuracies in the assessment of oral health behaviours and perceptions. Finally, while the study employed a standardized WHO assessment form, the exclusion of severely ill individuals may have led to an underestimation of the overall burden of oral health problems within these communities.

## 5 Conclusion

The oral health status of Irulars and Narikuravars of Villupuram district in Tamil Nadu is at stake. Narikuravas has more oral lesion compared to Irulas. The majority of Narikuravas and Irulas has high frequency of dental caries and periodontal disease. This study gives essential information to formulate the treatment schemes for the tribal community. A comprehensive state-wide study would be needed to obtain an accurate understanding of the oral health conditions and treatment needs.

Not Applicable.

## Human Ethics and consent to participate declaration

Consent to participate was obtained from all participants. For children below 14 years, the proxy consent was obtained from their parents/guardians.

## Authors contributions

Kalaivani V: Literature search, data collection, manuscript drafting.

Arthi Balasubramaniam: Data verification, data analysis, manuscript correction.

Meignana Arumugham: Manuscript correction.

## Funding

This research was self-funded

## Declaration of competing interest

The authors declare that they have no known competing financial interests or personal relationships that could have appeared to influence the work reported in this paper.
